# Antioxidant and Antimicrobial Evaluation and Chemical Investigation of *Rosa gallica* var. *aegyptiaca* Leaf Extracts

**DOI:** 10.3390/molecules26216498

**Published:** 2021-10-27

**Authors:** Ahmed S. Abdelbaky, Abir M. H. A. Mohamed, Salman S. Alharthi

**Affiliations:** 1 Department of Biochemistry, Faculty of Agriculture, Fayoum University, Fayoum 63514, Egypt; 2 Department of Agricultural Microbiology, Faculty of Agriculture, Fayoum University, Fayoum 63514, Egypt; amh05@fayoum.edu.eg; 3 Department of Chemistry, College of Sciences, Taif University, P.O. Box 11099, Taif 21944, Saudi Arabia; s.a.alharthi@tu.edu.sa

**Keywords:** *R. gallica* var. *aegyptiaca*, antioxidant activity, antimicrobial activity, plant extracts, HPLC-DAD, total phenolic content

## Abstract

*Rosa gallica* var. *aegyptiaca* is a species of flowering plant belonging to the Rosaceae family that plays an important role as a therapeutic agent for the treatment of specific types of cancer, microbial infections, and diabetes mellitus. This work presents the first report on the evaluation of the antioxidant and antimicrobial potential along with the phytochemical analysis of *Rosa gallica* var. *aegyptiaca* leaves. Five leaf extracts of hexane, chloroform, methanol, hydromethanol 80%, and water were prepared. Assessment of antioxidant activity was carried out via DPPH radical scavenging assay. Antimicrobial activity against five foodborne pathogenic bacteria—including *Listeria monocytogenes*, *Bacillus subtilis*, *Staphylococcus aureus*, *Escherichia coli*, and *Salmonella enteritidis*—and the fungus *Candida albicans*, was examined using the disc diffusion method. Total phenolic content and total flavonoid content were determined using the Folin–Ciocalteu reagent and aluminum chloride methods, respectively. Isolation, identification, and quantification of phenolic compounds were performed using HPLC-DAD analysis. Amongst the five leaf extracts that were investigated, hydromethanol 80% extract possessed the highest extraction yield, antioxidant activity, total phenolic content, and antimicrobial activity against all tested microbial strains. Moreover, this extract furnished six active phenolic compounds: gallic acid (1), (+) catechin (2), chlorogenic acid (3), (–) epicatechin (4), quercetin-3-*O*-α-d-(glucopyranoside) (5), and quercetin (6). This study provides an alternative utilization of *R. gallica* var. *aegyptiaca* leaves as a readily accessible source of natural antioxidants and antimicrobials in the food and pharmaceutical industries.

## 1. Introduction

Plants are powerful sources of potentially effective natural antioxidants and antimicrobial agents, and have attracted great interest recently. Several studies have been conducted in an attempt to discover natural antioxidant compounds that could potentially replace synthetic antioxidants [1]. Currently, synthetic antioxidants such as butylated hydroxytoluene (BHT, E321), tert-butylhydroquinone (TBHQ, E319), and butylated hydroxyanisole (BHA, E320) are commonly used in food for the prevention of oxidative deterioration [2]. It has been reported that these synthetic antioxidants are involved in some harmful side effects, such as the promotion of tumors, causing toxicity and cancer, as well as the wide rejection by consumers of synthetic food additives [3,4,5]. As a result, there is a need to search for novel and safe antioxidants derived from natural sources, which are more efficient and less hazardous. 

On the other hand, food poisoning is quite possibly the most widely recognized reason for illness and death caused by microorganisms in developing and third-world nations [6,7,8]. The consumption of foods contaminated with some microorganisms—particularly Gram (–) bacteria such as *Escherichia coli*, *Salmonella typhi*, and *Pseudomonas aeruginosa* [9,10], as well as Gram (+) bacteria such as *Staphylococcus aureus* and *Bacillus cereus* [11]—represents a significant health risk to humans. The survival and growth of microorganisms (especially bacteria) in foods causes spoilage, toxin formation, and quality deterioration in food products [12]. Due to the resistance that pathogenic bacteria and fungi possess against antibiotics, there is a lot of interest in finding new, healthier, safer, and potentially effective antimicrobial drugs from natural sources. In these contexts, natural plant extracts are used as antimicrobial agents for food poisoning diseases. These plant extracts and bioactive substances obtained from plant species can be utilized in folk medicine, and as a productive resource for such new medications [13]. Recently, many studies on antimicrobial activities using different plant extracts have revealed that the phenolic and flavonoid compounds found in plant extracts may also play a significant role in their antimicrobial action [13,14]. 

*R. gallica* var. *aegyptiaca* (Rosaceae) is considered one of the most widely important popular garden shrubs for the flavor, cosmetics, and fragrance industries [15]. Some *Rosa* species—such as *R. damascena*, *R. sempervirens*, and *R. villosa*—are commonly used for different therapeutic purposes, such as treatment of hemorrhoids, dyspepsia, or nephritis; as an expectorant, diuretic, stomachic, or tonic agent; for inflammations [16,17]; as an analgesic; to treat injuries; as a gastroprotective; or for certain types of cancer, microbial infections, diarrhea, or diabetes mellitus [18,19,20,21]—due to the presence of various phenolics, e.g., phenolic acids and flavonoids, terpenes, and essential oils in *Rosa* spp., which directly or indirectly contribute to their biochemical activities [22,23,24,25,26,27,28]. Moreover, some researchers found that various extracts and essential oils derived from several *Rosa* spp. possess strong antioxidant and antimicrobial activities [25,26,27,28,29,30]. 

Despite the popular use of *Rosa* species as therapeutic agents, to date there have been no data regarding their antioxidant and antimicrobial effects against foodborne pathogenic bacteria—including *Listeria monocytogenes*, *Bacillus subtilis*, *Staphylococcus aureus*, *Escherichia coli*, and *Salmonella enteritidis*, as well as the fungus *Candida albicans*—or the chemistry of *Rosa gallica* var. *aegyptiaca* leaf extracts. Thus, the present study aims to evaluate their antioxidant and antimicrobial activities, in addition to phytochemical analysis. 

## 2. Results and Discussion 

### 2.1. Qualitative Phytochemical Screening

Phytochemical screening of the dry powdered leaves of *R. gallica* var. *aegyptiaca* was carried out for the detection of various phytochemical constituents, and the results are presented in Table 1, which reveals the presence of saponins, triterpenoids, phenolics, tannins, flavonoids, alkaloids, glycosides, and carbohydrates, but no steroids.

### 2.2. Extraction Yields

The components of *R. gallica* var. *aegyptiaca* were extracted using solvents with increasing polarity: n-hexane (C_6_H_14_), chloroform (CHCl_3_), methanol (MeOH), hydromethanol 80% (MeOH/H_2_O 80%), and water (H_2_O). The product of extractable compounds (residues) relative to the weight of air-dried powdered leaves ranged from 1.6% (n-hexane extract) to 9.9% (hydromethanol extract) Table 2.

### 2.3. Antioxidant Activity via DPPH Free Radical Scavenging Assay

**Figure 1:**
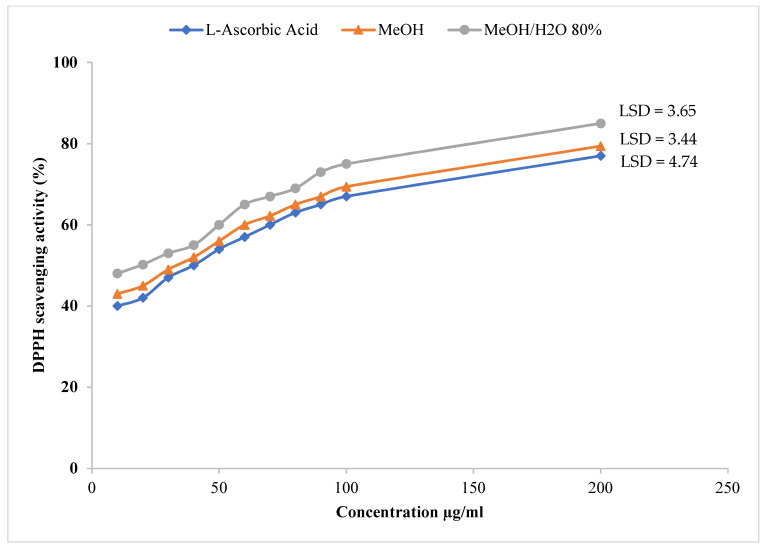
DPPH FRSA of MeOH/H_2_O 80% and MeOH leaf extracts of *R. gallica* var. *aegyptiaca*, compared with L-ascorbic acid (AA) (*n* = 3). LSD as a post hoc test at *p* ≤ 0.05 was used to separate the means of the treatments.

A DPPH assay at 50 µg/mL was used to determine the antioxidant activity of *R. gallica* var. *aegyptiaca* leaf extracts. The results (Table 2) are presented as inhibition percentage of free radical DPPH. As shown in Table 2, the highest free radical scavenging activity (FRSA) values were noted for MeOH/H_2_O 80% (97.2%), followed by MeOH (95.08%), while the lowest values were detected in extracts of C_6_H_14_ (20%), CHCl_3_ (15%), and H_2_O (10%). The variation in DPPH FRSA could be related to the variances in their secondary constituents—particularly phenolics [31,32]. The high antioxidant activity of the MeOH/H_2_O 80% and MeOH extracts was then used to estimate their IC_50_ values—the concentration of a substance required to inhibit 50% of the DPPH free radicals. The IC_50_ values of MeOH/H_2_O 80% and MeOH leaf extracts of *R. gallica* var. *aegyptiaca*, along with l-ascorbic acid (positive control), are presented in Table 2. As shown in the table, the highest DPPH FRSA was obtained in the MeOH/H_2_O 80% extract, with the lowest IC_50_ of 19.38 ± 0.38 µg/mL, followed by the MeOH extract, with 20.28 ± 0.97 µg/mL. These IC_50_ values of the MeOH/H_2_O 80% and MeOH were found to be stronger antioxidants than the methanol extract of white Rose, whose IC_50_ value was 43.8 µg/mL [33]. Moreover, the antioxidant activity of MeOH/H_2_O 80% and MeOH of *R. gallica* var. *aegyptiaca* on DPPH FRSA was found to be superior to that of *Rosa agrestis* ethyl acetate leaf extract (IC_50_ = 47.43 µg/mL) [26], and the MeOH/H_2_O 80% also showed a superior–excellent antioxidant activity compared with the conventional reference L-ascorbic acid (AA), with an IC_50_ of 21.30 µg/mL (Figure 1 and Table 2). Therefore, the MeOH/H_2_O 80% and MeOH extracts showed potent DPPH FRSA, indicating that *R. gallica* var. *aegyptiaca* leaves contain notable antioxidant compounds. These results indicate that both MeOH/H_2_O 80% extract and MeOH extract possess powerful antioxidant compounds, which may be responsible for their antioxidant activities, suggesting that this plant represents a promising source of natural antioxidants.

### 2.4. Total Phenolic and Flavonoid Contents

It is generally recognized that phenolic and flavonoid compounds play a direct role in plant biological activities, such as antioxidant [34] and antimicrobial [14] activity. Therefore, the TPC and TFC in the five different leaf extracts were determined. As presented in Table 2, the TPC and TFC of all leaf extracts differed significantly. The MeOH/H_2_O 80% (253.8 ± 1.26 mg GAE/g dried leaf extract) had the highest TPC level, followed by the MeOH (181.6 ± 0.83), while the TPC of the H_2_O, CHCl_3_, and C_6_H_14_ were the lowest (50.83 ± 1.25, 25.00 ± 3.10, and 20.00 ± 2.46 mg GAE/g of dried leaf extract, respectively). The TPC of the MeOH/H_2_O 80% was observed to be higher than the values stated by Bitis et al. [16] for *R. sempervirens* L. leaves (203.8 mg GAE/g), by Ozkan et al., [35] for *R. damascena* dried flower extracts (248.97 mg GAE/g), and by Nowak and Gawlik-Dziki [28] for *R. canina* var. *dumalis* leaves (15.2 mg GAE/g). These results are in accordance with those reported by Peschel et al. [36], who found that aqueous methanol (75%) is the most appropriate solvent for the extraction of phenolic compounds.

The TFC of *R. gallica* var. *aegyptiaca* leaf extracts ranged from 1.700 ± 0.22 to 54.48 ± 1.79 mg RE/g of dried leaf extract. As shown in Table 2, the highest TFC of 54.48 ± 1.79 was observed in MeOH, followed by MeOH/H_2_O 80% extract (41.02 ± 5.55 mg RE/g), whereas the CHCl_3_, C_6_H_14_, and H_2_O leaf extracts showed the lowest TFCs of 6.500 ± 1.50, 5.300 ± 1.25, and 1.700 ± 0.22 mg RE/g of dried extract, respectively. A similar finding that TFC in methanol extract is higher than in hydromethanol extract was observed by Tatke et al. [37]. The differences in the phytoconstituents may be explained by the use of extracting solvents that possess different natures and a variety of phenolic compounds. This perception leads to the inference that the potent antioxidant activity is due to the high amounts of soluble phenolic compounds in the most effective extracts.

### 2.5. HPLC-DAD Analysis

**Figure 2:**
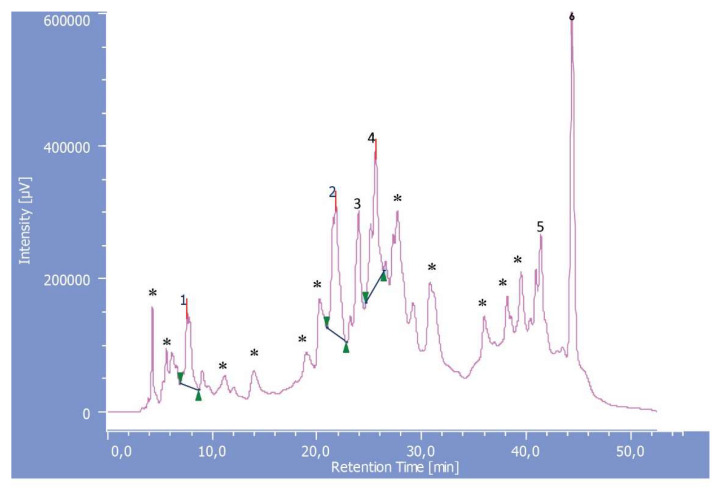
HPLC-DAD chromatogram of MeOH/H_2_O 80% extract of *R. gallica* var. *aegyptiaca* leaves. The figure shows the chromatographic separation of phenolic compounds based on the following conditions: Column, Zorbax C_18_, 5µm, 280 × 4.6 mm i.d.; detection at 350, 325, and 280 nm; flow rate, 0.5 mL/min; gradient elution system of methanol/water/formic acid; injected volume, 10 µL. Peaks: (1) gallic acid; (2) (+) catechin; (3) chlorogenic acid; (4) (–) epicatechin; (5) quercetin-3-*O*-α-d -(glucopyranoside); (6) quercetin; (*) unknown peaks.

HPLC-DAD analysis of MeOH/H_2_O 80% leaf extract furnished six phenolic compounds: gallic acid (1), (+) catechin (2), chlorogenic acid (3), (–) epicatechin (4), quercetin-3-*O*-α-d-(glucopyranoside) (5), and quercetin (6), which were identified by comparing their retention times and UV spectra to those of the standards, as shown in Figure 2 and Table 3, respectively. Also, the data of the HPLC-ESI-MS (Supplementary Materials, Figure S1) of the active fraction has confirmed the presence of these compounds.

Phenolics and flavonoids possess different antioxidant efficiency, depending on their structural conformation, number, and arrangement of the (-OH) groups and their localization in the structure [38,39,40]. Generally, both phenolic acids and other flavonoids such as flavonols and flavanols are considered to be effective hydrogen donors because of their carboxylic acid group and hydroxyl functional groups, which are easily ionized [41]. In this study, phenolic acids such as gallic and chlorogenic acids (peaks 1 and 3) were identified at amounts of 1.7 and 1.8 mg/g, respectively. In addition to other flavonoids, (+) catechin, (–) epicatechin, and quercetin-3-*O*-α-d-(glucopyranoside) (peaks 2, 4, and 5, respectively) were identified in acceptable amounts, while quercetin (peak 6) was present in a high amount (19.8 mg/g), and was considered to be the main component.

The MeOH/H_2_O 80% extract from *R. gallica* var. *aegyptiaca* leaves contained the highest concentration of flavonol (quercetin), which may be very interesting, since this compound has been correlated with various biological effects, including antioxidant, anti-inflammatory, anticarcinogenic, antiviral, antibacterial, antifungal, antitumor, antiallergenic, and antithrombotic activities [42,43,44,45].

### 2.6. Antimicrobial Activity

Five different leaf extracts were investigated at a concentration of 10 mg/disc for their antimicrobial activity against five food poisoning bacteria—namely, *L. monocytogenes*, *B. subtilis*, *S. aureus*, *E. coli*, *S. enteritidis*—and the fungus *C. albicans* as well, using the disc diffusion technique. The antimicrobial activity of these leaf extracts was assessed by evaluating the diameter of the inhibition zone (DIZ, mm), as recorded in Table 4. All solvents, as negative controls, had no inhibitory effect against any of the tested microorganisms. The results in Table 4 show that the MeOH/H_2_O 80% and MeOH extracts exhibited inhibitory effects against all tested bacteria as well as the fungus, while the H_2_O extract was only capable of inhibiting the growth of all tested bacteria. These results are in agreement with the findings of Tatke et al. [37], who found that *Rosa damascena* methanol extract exerted antimicrobial activity against all tested microorganisms, while water extract did not show any ability as an antifungal (i.e., *C. albicans*). However, the C_6_H_14_ and CHCl_3_ extracts were active only against *E. coli*, and these results are consistent with the findings of Halawani [46].

The active extracts had varying degrees of antimicrobial potential against the tested microbial strains. The MeOH/H_2_O 80% extract also showed a superior–excellent antibacterial potency compared with the antibiotic gentamycin. The MeOH/H_2_O 80% extract was found to be the most efficient, and has a broad antibacterial spectrum when tested against both Gram (+) and Gram (–) bacteria, as well as fungi. The susceptible bacteria for the MeOH/H_2_O 80% followed the sequence *E. coli* > *B. subtilis* > *S. enteritidis* > *S. aureus* > *L. monocytogenes*, with the zone of inhibition ranging from 17 mm to 25 mm. These results are of great importance, especially with respect to *S. aureus* and *S. enteritidis*, as they are well known for their antibiotic resistance and for producing a variety of enterotoxins that induce various forms of enteritis and septicemia. The antimicrobial activity of the MeOH/H_2_O 80% of *R. gallica* var. *aegyptiaca* was obviously related to its phenolic and flavonoids components, since HPLC-DAD analysis proved that this extract possesses an appropriate amount of phenolic acids, flavonol, and flavan-3-ol, which are responsible for the activities shown. From these results, *R. gallica* var. *aegyptiaca* leaf extracts—particularly the MeOH/H_2_O 80%—could be a promising natural preservative against foodborne pathogens for the industry of food production.

### 2.7. Antimicrobial Activity of the Identified Phenolic Compounds from MeOH/H_2_O 80% Extract of R. gallica *var.* aegyptiaca Leaves

The antimicrobial activity of the identified phenolic compounds was investigated against the tested microbial strains. The results in Table 5 show that all identified phenolics had antimicrobial effects against all tested microorganisms to varying degrees. Table 5 also shows that the major phenolic component, quercetin, exhibited the highest lethal effect against all examined microbial strains. In addition, it possessed strong antibacterial and antifungal activities against tested bacterial and fungal strains, with a zone of inhibition (ZI) ranging from 24 to 30 mm and 17 mm, respectively—more potent than those of the standard antibacterial and antifungal agents, i.e., gentamycin and fluconazole.

It should be noted that there have been no previous studies published on the identification of these compounds from the leaves of *R. gallica* var. *aegyptiaca*. Thus, this is the first study on the identification of these antimicrobial phenolics from the leaves of this plant. It has been reported that various isolated flavonoid compounds from plant extracts have potent antimicrobial activity [47,48,49]. The obtained results indicate that these phenolics—especially quercetin—may be used as lead compounds for the development of new natural antimicrobial agents.

## 3. Materials and Methods

### 3.1. Chemicals

The reference compounds and solvents that were used for the analytical procedures and the extractions—i.e., gallic acid, rutin, L-ascorbic acid, n-hexane, chloroform, methanol, Folin–Ciocalteu reagent, and 1,1-diphenyl-2-picrylhydrazyl (DPPH)—were purchased from Sigma-Aldrich (St. Louis, MO, USA); aluminum chloride and sodium carbonate were of analytical grade.

### 3.2. Plant Material

Fresh leaves of *Rosa gallica* var. *aegyptiaca* were collected from Orman Botanical Garden, Giza, Egypt, in August 2020. Taxonomic identification of the plant was established by Professor Dr. T. Labeb at the Horticulture Research Institute, Giza, Egypt. A voucher specimen (No. 125) was deposited in the herbarium of the Biochemistry Department, Faculty of Agriculture, Fayoum University, Fayoum, Egypt. The leaves were washed gently with distilled water to eliminate dust and soil, air-dried in the shade, and then crushed to powder using a laboratory mill through a 24-mesh sieve. Powdered materials were stored in an airtight bottle at room temperature (28 ± 2 °C) and protected from light for further use.

### 3.3. Extraction and Isolation

The dried powder (100 g/500 mL) of *R. gallica* var. *aegyptiaca* leaves was taken and extracted with n-hexane (C_6_H_14_), chloroform (CHCl_3_), methanol (MeOH), hydromethanol (MeOH/H_2_O 80%), and water (H_2_O) individually for three successive days, at room temperature (28 ± 2 °C), with persistent shaking. The resulting extracts were filtered over a Whatman filter paper No. 1, and then the combined extract was evaporated at 45 °C to dryness using a rotary evaporator (R300, BUCHI Labortechnik AG, Flawil, Switzerland). All leaf extracts were used separately for screening the antioxidant and antimicrobial activities. The percentage yield was calculated using the following equation:

Extract yield (%) = (W_1_/W_2_) × 100 (1)

where W_1_ is the weight of extracted plant residues in grams, and W_2_ is the weight of air-dried powdered leaves in grams.

For the isolation of phenolics and flavonoids from the MeOH/H_2_O 80% extract, a known weight of the hydromethanolic extract (3.5 g) was chromatographed through a silica gel column (2.7 cm × 60 cm, 100 g) using CHCl_3_:MeOH (100:0→0:100, *v*/*v*) and monitored via TLC with a suitable system (CHCl_3_: MeOH, 70:30, *v*/*v*). The eluates were combined on the basis of similarity of TLC profiles to afford 5 fractions (A–E), and were then tested for antioxidant and antimicrobial activity. The active fraction (C, 1.15 g) was then chromatographed via HPLC-DAD on an RP-18 (4.6 × 280 mm, 5 µm), using methanol and water/formic acid (90:10, *v*/*v*) as a mobile phase (flow rate 0.5 mL/min.), to yield compounds 1 (1.7 mg), 2 (2.9 mg), 3 (1.8 mg), 4 (2.6 mg), 5 (0.5 mg), and 6 (19.8 mg).

### 3.4. Qualitative Phytochemical Screening

Phytochemical detection was carried out to identify the various phytoconstituents present in the leaves of *R. gallica* var. *aegyptiaca*, using standard phytochemical methods [50,51,52].

### 3.5. DPPH Radical Scavenging Activity

The ability to scavenge free radicals of all leaf extracts from *R. gallica* var. *aegyptiaca* was measured via 1,1-diphenyl-2-picrylhydrazyl (DPPH) free radical assay based on the method described by Brand-Williams et al. [53].

### 3.6. Total Phenolic Content Determination (TPC)

The TPs in the leaf extracts of *R. gallica* var. *aegyptiaca* were determined using the Folin–Ciocalteu reagent [54].

### 3.7. Total Flavonoid Content Determination (TFC)

The aluminum chloride method described by Lamaison and Carnet [55] was used to determine TFC in the leaf extracts of *R. gallica* var. *aegyptiaca*.

### 3.8. HPLC-DAD Analysis

Isolation and identification of main phenolics were carried out using an HPLC 1100 coupled with a diode array detector (DAD) (model G1315B DAD system; Agilent Technologies, Santa Clara, CA, USA.). The reversed stationary phase employed was a Zorbax C_18_ column (280 × 4.6 mm, internal diameter (i.d.), particle size 5 µm; Agilent Technologies) with a pre-column C_18_, 5µm (4 × 2 mm i.d., Phenomena, Castel Maggiore, Bologna, Italy). The following gradient system was used with methanol (eluent A) and water/formic acid (90:10, *v*/*v*) (eluent B): the flow rate was 0.5 mL/min, the sample injection was 10 µL, and the gradient program was initiated from 5%A, 95%B (10 min); 40%A, 60%B (20 min); 80%A, 20%B (50 min); 5%A, 95%B (55 min); analyses were stopped after 70 min, followed by washing and then re-equilibration of the column. The elution protocol and flow rate were both monitored using an LC-Chem-Station 3D software program (Hewlett-Packard, Palo Alto, CA, USA). DAD was between 250 and 650 nm, and absorbance was recorded at 350, 325, and 280 nm. Identification of phenolic compounds was achieved by comparing the retention times and diode array UV spectra with those of pure standards. The external standard technique was used to quantify phenolics via the integration of the individual peaks.

### 3.9. Antimicrobial Activity

#### 3.9.1. Microbial Strains

The antimicrobial activity of *R. gallica* var. *aegyptiaca* leaf extracts was tested against five strains of human pathogenic bacteria (i.e., foodborne diseases), including three Gram (+) bacteria (*Listeria monocytogenes* ATCC 15313, *Bacillus subtilis* ATCC 7030, and *Staphylococcus aureus* ATCC 8095) and two Gram (–) bacteria (*Escherichia coli* ATCC 25922 and *Salmonella enteritidis* ATCC 13076). Antifungal activity was tested using *Candida albicans*. These strains were obtained from the Agricultural Microbiology Department, Faculty of Agriculture, Fayoum University (Fayoum, Egypt). The stock cultures of bacteria were maintained on Luria broth (LB) slants, and Candida yeast on potato dextrose agar (PDA) slants, at 4 °C.

#### 3.9.2. Inoculums Preparation

Prior to assay, colonies of the tested bacteria and yeast strain were picked from sub-cultured overnight slants freshly prepared and transferred to 5 mL LB broth medium for bacteria—or PDA for yeast—and incubated at 37 °C for 24–48 h in the case of pathogenic bacteria, and at 30 °C for 48–72 h in the case of *Candida* strain. The density of all culture suspensions was adjusted to the value of the standard equivalent 0.5 McFarland by measurement with the spectrophotometer. The different broth cultures were used to inoculate Petri dishes prior to the antimicrobial assay of plant extracts.

#### 3.9.3. Antimicrobial Assay

Antimicrobial assay of each leaf extract against the tested microbial strains was performed using agar disc diffusion assay according to the method described by Bauer et al. [56]. The sterile filter paper discs (8 mm in diameter) were impregnated with 40 µL of each plant extract (10 mg/40 µL) after sterilization by filtration through a 0.22 μm membrane filter. The dried sterile filter paper discs loaded with leaf extract of *R. gallica* var. *aegyptiaca* were placed on the surface of the inoculated plates in triplicate. The inoculated plates were stored in the fridge at 4 °C for two hours to allow diffusion of leaf extracts, and then incubated as previously described. After this time, the bioactivity was assessed by measuring the diameter of the inhibition zone (DIZ) formed around each disc in mm. As a negative control, a disc containing only 40 μL of solvent (without test extract) was employed. For bacteria and fungi, positive controls were gentamycin and fluconazole, respectively. All assays were performed in triplicate, and means (±S.D.) were calculated using the Microsoft Excel program.

### 3.10. Statistical Analysis

All experiments were expressed as mean ± SD in triplicate. General linear model (GLM) procedures were used to analyze the data. Differences at *p* ≤ 0.05 were considered significant using Statistical Package for the Social Sciences (SPSS) software (SPSS, 2019).

## 4. Conclusions

In the current study, the antioxidant and antimicrobial potential of five extracts—including n-hexane, chloroform, methanol, methanol/water 80%, and water—from the leaves of *R. gallica* var. *aegyptiaca* was evaluated, alongside phytochemical analysis. Among the five leaf extracts, hydromethanol 80% extract possessed the highest extraction yield and total phenolic content, and also exhibited very high antioxidant and antimicrobial activities against all tested microbial strains. Furthermore, six active phenolic compounds—gallic acid (1), (+) catechin (2), chlorogenic acid (3), (–) epicatechin (4), quercetin-3-*O*-α-d-(glucopyranoside) (5), and quercetin (6)—were identified and quantified from this leaf extract via HPLC-DAD analysis for the first time. Thus, the use of the leaves of this plant as natural antioxidants and antimicrobial sources appears to be a viable alternative to synthetic antioxidants and antimicrobials to alleviate human health hazards associated with the use of these synthetic compounds.

## Figures and Tables

**Table 1:** Qualitative phytochemical screening of *R. gallica* var. *aegyptiaca* leaves.

Constituent	Detection Test	Result
Saponins	Foam test	+
Steroids	Liebermann–Burchard test	–
Triterpenoids	Salkowski reaction	+
Phenolic compounds and tannins	Ferric chloride test	+
Flavonoids	Lead acetate test	+
Alkaloids	Wagner’s tests	+
Glycosides	Keller–Kiliani test	+
Carbohydrates	Molisch’s test	+

(+): present; (–): absent.

**Table 2:** Extraction yield, antioxidant activity (DPPH inhibition percentage), IC_50_, total phenolics (TPs), and total flavonoids (TFs) of *R. gallica* var. *aegyptiaca* leaf extracts.

Extract	Extract Yield (%)	Inhibition Percentage (50 µg/mL)	IC_50_ (µg/mL)	TPs (mg GAE ^g^/g Extract)	TFs (mg RE ^r^/g Extract)
C_6_H_14_	1.6	20 ± 1.0 ^c^		20.00 ± 2.46 ^e^	5.300 ± 1.25 ^d^
CHCl_3_	6.5	15 ± 85 ^d^		25.00 ± 3.10 ^d^	6.500 ± 1.50 ^c^
MeOH	9.2	95.08 ± 0.33 ^b^	20.28 ± 0.97 ^b^	181.6 ± 0.83 ^b^	54.48 ± 1.79 ^a^
MeOH/H_2_O 80%	9.9	97.20 ± 0.25 ^a^	19.38 ± 0.85 ^a^	253.8 ± 1.26 ^a^	41.02 ± 1.55 ^b^
H_2_O	4.1	10 ± 1.1 ^e^		50.83 ± 1.25 ^c^	1.700 ± 0.22 ^e^
L-Ascorbic Acid			21.30 ± 0.55 ^c^		

Values are expressed as mean ± SD, *n* = 3. Means within each column with different letters (^a–e^) are significantly different (*p* < 0.05); ^g^ GAE: gallic acid equivalents; ^r^ RE: rutin equivalents. Means sharing the same letter for each parameter are not significantly different according to LSD as a post hoc test at *p* ≤ 0.05.

**Table 3:** Identification and quantification of the phenolic compounds present in MeOH/H_2_O 80% leaf extract of *R. gallica* var. *aegyptiaca* via HPLC-DAD.

Peak	Rt (min)	Compound	mg/g
1	7.56	Gallic acid	1.7
2	21.77	Catechin	2.9
3	23.68	Chlorogenic acid	1.8
4	25.56	Epicatechin	2.6
5	41.30	quercetin-3-*O*-α*-*d-(glucopyranoside)	0.5
6	44.33	Quercetin	19.8

**Table 4:** Evaluation of in vitro antimicrobial activity of *R. gallica* var. *aegyptiaca* leaf extracts.

Extract	Antimicrobial Activity					
	Diameter of Inhibition Zones (mm)					
	Gram (+) Pathogenic Bacteria			Gram (–) Pathogenic Bacteria		Fungi
	*L. monocytogenes*	*B. subtilis*	*S. aureus*	*E. coli*	*S. enteritidis*	*C. albicans*
C_6_H_14_	-	-	-	13 ± 1.53 ^f^	-	-
CHCl_3_	-	-	-	16 ± 1.00 ^d^	-	-
MeOH	16 ± 2.00 ^ab^	19 ± 1.00 ^b^	17 ± 1.15 ^b^	24 ± 1.15 ^ab^	19 ± 1.00 ^a^	10 ± 1.10 ^c^
MeOH/H_2_O 80%	17 ± 0.58 ^a^	20 ± 0.58 ^a^	17 ± 1.53 ^b^	25 ± 2.52 ^a^	19 ± 1.00 ^a^	11 ± 1.50 ^b^
H_2_O	12 ± 0.76 ^c^	12 ± 1.73 ^c^	19 ± 0.58 ^a^	20 ± 2.00 ^c^	18 ± 1.15 ^b^	-
Gentamycin (10 mg)	15 ± 1.00 ^b^	12 ± 1.15 ^c^	16 ± 0.58 ^c^	15 ± 2.08 ^e^	13 ± 1.00 ^c^	n.d.
Fluconazole (10 mg)	n.d.	n.d.	n.d.	n.d.	n.d.	14 ± 1.05 ^a^

(–) No inhibition; values are means (*n* = 3). The concentration of each leaf extract was 10 mg/disc. n.d.: not determined. Means sharing the same letters (^a–f^) for each column are not significantly different according to LSD as a post hoc test at *p* ≤ 0.05.

**Table 5:** Antimicrobial activity of the identified phenolic compounds, represented by diameter of inhibition zone (DIZ, mm).

Compounds	Antimicrobial Activity					
	Diameter of Inhibition Zones (mm)					
	Gram (+) Pathogenic Bacteria			Gram (–) Pathogenic Bacteria		Fungi
	*L. monocytogenes*	*B. subtilis*	*S. aureus*	*E. coli*	*S. enteritidis*	*C. albicans*
Gallic acid	17 ± 1.10 ^b^	16 ± 1.13 ^c^	18 ± 1.57 ^c^	20 ± 0.45 ^b^	18 ± 1.30 ^b^	11 ± 1.10 ^e^
Catechin	16 ± 0.95 ^c^	18 ± 1.50 ^b^	16 ± 0.20 ^e^	17 ± 1.16 ^c^	15 ± 0.56 ^c^	10 ± 1.35 ^ef^
Chlorogenic acid	15 ± 0.35 ^d^	15 ± 0.50 ^cd^	17 ± 0.13 ^d^	16 ± 0.95 ^d^	14 ± 1.65 ^d^	11 ± 1.00 ^e^
Epicatechin	16 ± 0.55 ^c^	14 ± 1.02 ^d^	17 ± 0.50 ^d^	15 ± 1.40 ^e^	14 ± 0.33 ^d^	12 ± 0.85 ^d^
Quercetin-3-glucoside	17 ± 1.02 ^b^	18 ± 0.40 ^b^	20 ± 1.16 ^b^	19 ± 1.22 ^bc^	17 ± 0.57 ^bc^	15 ± 1.15 ^b^
Quercetin	28 ± 0.57 ^a^	25 ± 0.87 ^a^	24 ± 1.12 ^a^	30 ± 1.18 ^a^	26 ± 0.56 ^a^	17 ± 0.46 ^a^
Gentamycin (10 µg)	15 ± 1.00 ^d^	12 ± 1.15 ^e^	16 ± 0.58 ^e^	15 ± 2.08 ^e^	13 ± 1.00 ^e^	n.d.
Fluconazole (10 µg)	n.d.	n.d.	n.d.	n.d.	n.d.	14 ± 1.05 ^c^

n.d.: Not determined. Means sharing the same letters (^a–f^) for each column are not significantly different according to LSD as a post hoc test at *p* ≤ 0.05.

## Data Availability

The data presented in this study are available in this article.

## Supplementary Materials

The following is available online, Figure S1: HPLC-ESI-MS spectra.

## Sample Availability

Samples of the compounds are not available from the authors.
