# Supplementary material for: Antioxidant and Antimicrobial Evaluation and Chemical Investigation of Rosa gallica var. aegyptiaca Leaf Extracts
Source: Molecules. 2021 Oct 27;26(21):6498. doi: 10.3390/molecules26216498 (PMC8587625; doi:10.3390/molecules26216498)
Supplement: Supplementary file 1 [file molecules-26-06498-s001.zip › molecules-1417900-supplementary.pdf]

HPLC-ESI-MS methods

Analyses were performed using a HP1100 series HPLC instrument (quaternary pump and degasser, column compartment, and auto-sampler) and an LCQ ADVANTAGE MAX mass spectrometer from Thermo Finnigan. ''Xcalibure 1.4'' software. Chromatographic separation was carried out at 30 °C. on Xterra C18 column (250 × 4.6 mm, particle size 5μm). The mobile phase consisted of 5% (v/v) methanol in water (eluent A) and 95% water [0.05% formic acid] (eluent B) at zero time. The flow rate was 0.5ml/min and the gradient programme was optimized as follows: 15% A and 85% B (10 min); 40% A and 60% B (20 min); 80% A and 20% B (55 min); 5% A and 95% B (65 min). Total run time was 65 min. The injection volume for the sample was 5 μl. Monitoring was performed at 280 nm. The HPLC was coupled to a mass spectrometer (MS) (Agilent, Palo Alto, CA) equipped with an electrospray ionization (ESI) source operating at the negative and positive ionization mode. Ionization parameters were as follows: Source voltage; 4.5 KV, source current; 100 uA, capillary voltage; 3 V, capillary temperature; 200 °C, sheath gas flow; 60 arb., Helium was used as a collision gas at a pressure of 2.5 to 3.5 mTorr, the collision energy was set to 25 eV, tube lens offset; 5 V, octapole RF amplifier 500 Vp-p, octapole offset (1); −2.5 V, octapole offset (2); 7 V, entrance lens; -20 V, and interoctapole lens voltage; -40 V. Identities of the compounds were obtained by matching their molecular ions (m/z) obtained by HPLC–ESI–MS and HPLC–ESI–MS/MS with literature data.

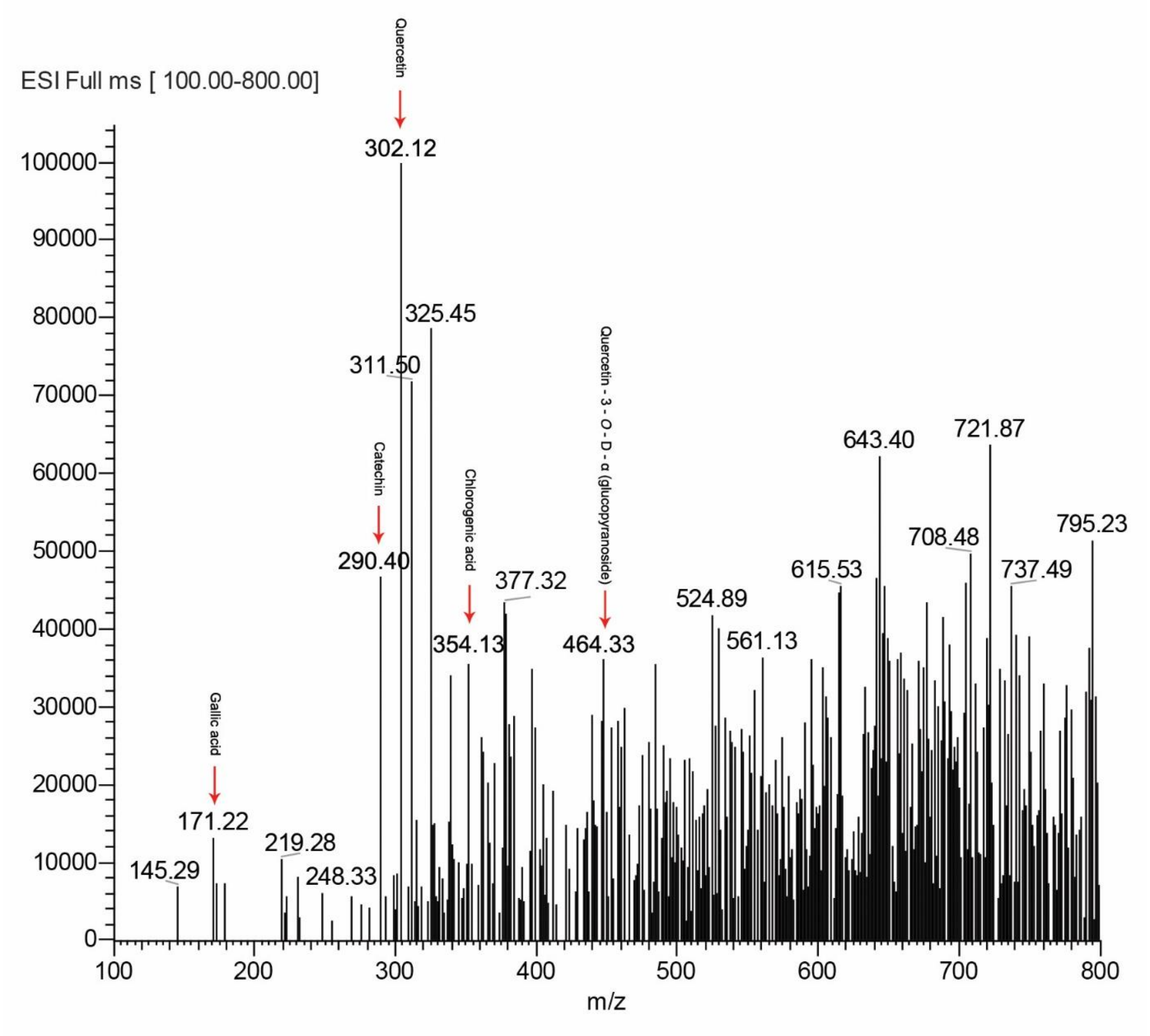

Figure S1. *HPLC-ESI-MS* spectra of active fraction No. C
